# Effects of Fatigue Running on Joint Mechanics in Female Runners: A Prediction Study Based on a Partial Least Squares Algorithm

**DOI:** 10.3389/fbioe.2021.746761

**Published:** 2021-09-24

**Authors:** Wenjing Quan, Feng Ren, Datao Xu, Fekete Gusztav, Julien S Baker, Yaodong Gu

**Affiliations:** ^1^ Faculty of Sports Science, Ningbo University, Ningbo, China; ^2^ Savaria Institute of Technology, Eötvös Loránd University, Szombathely, Hungary; ^3^ Centre for Health and Exercise Science Research, Department of Sport and Physical Education, Hong Kong Baptist University, Hong Kong, China

**Keywords:** joint work, kinetics, partial least-squares regression, fatigue, prolonged running

## Abstract

**Background:** Joint mechanics are permanently changed using different intensities and running durations. These variations in intensity and duration also influence fatigue during prolonged running. Little is known about the potential interactions between fatigue and joint mechanics in female recreational runners. Thus, the purpose of this study was to describe and examine kinematic and joint mechanical parameters when female recreational runners are subject to fatigue as a result of running.

**Method:** Fifty female recreational runners maintained running on a treadmill to induce fatigue conditions. Joint mechanics, sagittal joint angle, moment, and power were recorded pre- and immediately post fatigue treadmill running.

**Result:** Moderate reductions in absolute positive ankle power, total ankle energy dissipation, dorsiflexion at initial contact, max dorsiflexion angle, and range of motion of the joint ankle were collected after fatigue following prolonged fatigue running. Knee joint mechanics, joint angle, and joint power remained unchanged after prolonged fatigue running. Nevertheless, with the decreased ankle joint work, negative knee power increased. At the hip joint, the extension angle was significantly decreased. The range motion of the hip joint, hip positive work and hip positive power were increased during the post-prolonged fatigue running.

**Conclusion:** This study found no proximal shift in knee joint mechanics in amateur female runners following prolonged fatigue running. The joint work redistribution was associated with running fatigue changes. As for long-distance running, runners should include muscle strength training to avoid the occurrence of running-related injuries.

## Introduction

Running can be considered one of the most popular recreational physical activities worldwide that promote aerobic capacity and reduce the risk of cardiovascular disease (Chakravarty et al., 2008; Stamatakis and Chaudhury, 2008; Dempster et al., 2021). Running-related injuries have been identified as a common overuse injury in competitive and recreational runners. As a cyclical movement, long-distance running can cause overuse-related injuries due to the extended heavy impacts between the lower limb and the ground. It has been shown that the risk of running-related injury has increased from 6.9 to 92.4% per 1,000 h of running (Van Gent et al., 2007; Videbæk et al., 2015). In addition, female runners are subjected to a higher risk of overuse running injuries than male runners (Taunton et al., 2002; Boling et al., 2010). However, the mechanism of female running joint work distributed for fatigue running was still unclear.

Recently, some studies have focused on joint work distribution after long-distance running. Previous investigations have reported that the ankle energy generation significantly decreased in recreational runners compares to competitive runners. With an increase in running distance, the positive work contribution could shift from distal (ankle) to proximal (knee, hip) joints. The possible interpretation of this phenomenon is the following: runners who are subjected to fatigue due to intensive sports activities such as running, the ankle, plantar flexors might cause the ankle energy generation and ankle joint moment reduced (Sanno et al., 2018). This evidence shows that a redistribution occurs in the joint work, which can be considered as a primary factor in improving the metabolic cost during prolonged running. Simultaneously, the muscular capacity of competitive runners was significantly greater than the recreational runners, which might result in less plantarflexor after fatigue running. Evidence has demonstrated that the foot and ankle play an essential role during running, constituting more than 50% of the joint absorption and joint energy generation (Kelly et al., 2018; Cigoja et al., 2019). Interestingly, a previous study found that well-trained rearfoot strike runners did not show a proximal positive joint work shift followed by prolonged running (Melaro et al., 2021). It is worth mentioning that different running strike pattern designs were used in the study, which may have caused different results. However, there is little research about joint work in female runner’s fatigue running biomechanics. Future studies should consider the type of sex and distance when calculating the changes in joint work following fatigue induced from prolonged running (Melaro et al., 2021).

Additionally, fatigue running was the main factor that modify running biomechanics parameters. (Derrick et al., 2002). In 1999, Dutto et al. reported that after fatigue running, dorsiflexion at heel contact was more reduced than pre-fatigue; in 1981, Elliot et al. showed an increase in rearfoot motion after prolonged running, possibly due to fatigue (Elliot and Ackland, 1981; Dutto et al., 1997). Generally, fatigue might lead to a decline in the motor control ability of the musculoskeletal system and increase the risk of sports injuries (Lieber, 2018; Zheng, 2021). With respect to kinematic parameters, previous studies have pointed out that impact acceleration, trunk tilt, and ankle eversion angle were increased after prolonged running due to fatigue (Mizrahi et al., 2000). For example, after exhaustive running, the plantarflexion moment and external rotation moments were decreased (Benson and O’Connor, 2015; Hashish et al., 2016). Simultaneously, the knee and hip angular abduction impulses were significantly increased (Willson et al., 2015).

As for the running kinematics variables, it has been shown that the ankle and knee initial contact angle was crucial for joint stability (Bazuelo-Ruiz et al., 2018). Regarding IC (initial foot contact), contradictive studies have been published, where some authors claimed that the ankle angle is linearly influenced by joint absorption during running (Breine et al., 2017), while others stated that the connection was nonlinear (Chambon et al., 2015). There is limited research on how the initial angle affects joint work. Bastiaan Breine et al. found that the foot angle at initial contact during the rearfoot strike had the highest correlation with the vertical instantaneous loading rate (VILR) (Breine et al., 2017). This indicates that a greater foot angle or more pronounced rearfoot strike corresponded with a lower vertical instantaneous loading rate (VILR). Furthermore, following exhaustive treadmill running, knee flexion at foot contact was significantly increased (Derrick et al., 2002). Therefore, lower limb kinematics' modification following fatigue-induced running was associated with ankle initial foot contact and joint energy.

To the best of our knowledge, little work has been done on the effects of fatigue on joint work during the impact phase of long-distance running. Thus, the purpose of this study was to describe and examine kinematic and joint mechanical parameters when female recreational runners were subjected to fatigue after long-distance running. The analysis was carried out using the Partial Least Square Algorithm (PLSR) to investigate if a linear relationship existed between the initial joint angle, ankle joint work, and knee joint work. Our first hypothesis was that ankle work would decrease due to fatigue after prolonged running. Our second hypothesis was that joint work would have a greater relationship with the initial angle of the ankle and knee.

## Materials and Methods

### Participants

Fifty female recreational runners (23.89 ± 1.27 years, 65.39 ± 22.47 kg, 163.22 ± 15.01 cm) were recruited from the university running clubs, while flyers were distributed around the university campus for this investigation. Participants were screened to include individuals aged between 18 and 27 years, ran between 5 and 10 km per week, and did not have any low limb musculoskeletal injuries in the previous 6 months prior to data collection. All participants were rearfoot strikers and without any vigorous exercise 24 h before data capture. All subjects provided their signed informed consent, while ethical approval was obtained from the Sports Science Faculty at Ningbo University.

### Instrumentation

The kinematic data was acquired using a British-made Vicon infrared 3D motion capture system (Oxford Metric Ltd., Oxford, United Kingdom), including eight high-speed infrared cameras with Nexus analysis software. The sampling frequency for this study was 200 Hz. Ground reaction force (GRF) data were measured using a 90*58*10 cm force platform at 1000 Hz (9281B, Kistler Instruments AG, Switzerland). During the test process, heel strike and toe-off phases were defined when the vertical GRF crossed a 30 N threshold (Maas et al., 2018). Kinematic and kinetic data were collected simultaneously before the running started and when the person reached the point of exhaustion or fatigue. This point was detected by a heart rate monitor (Polar RS100, Polar Electro Oy, Woodbury, NY, United States), which was compulsory for all participants while running on the motorized treadmill (h/p/cosmos sports and medical GmbH, Germany). The subjects used conventional running shoes during the running experiments. Thirty-six retroreflective markers, with 14 mm-diameter, were attached to the right and left lower extremities to define the ankle, knee, and hip joints using a 6DOF market set flowing the previous study (Zhou et al., 2021).

### Running Fatigue Protocol

Prior to data collection, subjects were familiarized with the running protocol and the Borg Scale RPE 6–20. The Borg Scale RPE 6–20 and heart rate monitor were used to record subjective fatigue and heart rate changes during the running intervention. First, the subjects warmed up at 6 km/h for 3 min. Then, the operator increased the treadmill speed to 14.4 km/h. Subjects were required to run at 14.4 km/h on the treadmill until they could not continue. They were then considered in a fatigued state. Fatigue was defined when all the following conditions were met: 1) the heart rate of the participants reached 90% maximum heart rate of their age-calculated maximum heart rate (HRmax = 220-age), 2) the participants could not continue running, and 3) a rating on the Borg scale exceeded RPE > 17 (very hard) (Koblbauer et al., 2014).

### Experimental Protocol

There are two testing sessions in this study, which include the pre-fatigue and post-fatigue protocols. Before the test, all subjects had to wear uniform pants, T-shirts, socks, and running shoes. They used to jump and running activities for the warm-up session. In the pre-fatigue section, the participants ran through the force plate across a 15 m runway at 3.3 m/s to capture the kinematic and ground reaction force data. All participants performed the fatigue intervention on the treadmill in the post-fatigue section and then immediately ran over the force plate at 3.3 m/s to capture the kinematics and ground reaction force. For each subject, six successful running trials were collected. Using the speed measuring instrument (Smart speed, Fusion Sport Inc., Burbank, CA, United States) to control every subject running speed at 3.3 m/s. A successful trial was defined when a participant ran through the force platform using the right foot and the running speed was 3.3 m/s ±0.05.

### Data Analysis

#### Sagittal Plane Kinematics

The kinematic data was preprocessed using Vicon Nexus software, capturing a full running stance phase, completing any missing mark points, and removing any incorrect or redundant mark points during the process. After preprocessing, the biomechanical data was imported into Visual3D software (v6; C-Motion, Inc., Germantown, MD, United States) for processing and calculation. The kinematic and kinetic data were processed using a fourth-order Butterworth low-pass filter with cutoff frequencies of 15 and 50 Hz, respectively (Bezodis et al., 2008). Joint angles, joint moments, joint power, and joint work were normalized to the gait cycle over 101-time points. Ankle, knee and hip angles were calculated using Cardan angles in the sagittal plane (positive-flexion/dorsiflexion; negative-extension/plantarflexion) (Quan et al., 2021).

#### Joint Kinetics

The joint moments, including the maximum moment values of the ankle, knee, and hip joints, were calculated using an inverse dynamics approach. Joint power, including the maximum power values of the ankle, knee, and hip joints, was defined as the dot product of the joint moment and the angular velocity. The ankle joint dorsiflexion moment, knee joint flexion moment, and hip flexion moment are positive (+), and the corresponding ankle joint plantarflexion moment and hip joint extension moment are negative (−). The positive value (+) of the ankle, knee, and hip joint power indicates energy production. The negative value (−) of the ankle, knee and hip joint power indicates the energy absorption of the ankle, knee, and hip joints.

$$P_{j}=M_{j}\cdot \omega_{j}$$

*M*_*j*_ is the joint moment of the ankle, knee, and hip joint, while *ω*_*j*_ is the joint angular velocity of the ankle, knee, or hip. The joint work is obtained by integrating the joint power over time. In this paper, the trapezoidal method was used for numerical integration. Energy generation (*E*_*g*_) or energy absorption (*E*_*a*_) was calculated by the integral of the positive and negative areas of joint angular power at a time using a custom program over the stance phase in MATLAB (Version: R2019a, The MathWorks, Natick, MA, United States). Total joint work was calculated by integrating the joint power time curves over the stance phase, respectively (Koblbauer et al., 2014; Xu et al., 2021).

$$E_{i}=W_{i}=\int_{t1}^{t2}P_{j}\cdot \mathrm{dt}=\int_{t1}^{t2}M_{j}\cdot \omega_{j}\cdot \mathrm{dt}$$

Where *i* = *a* as absorption or *g* as generation, *t*_*1*_ to *t*_*2*_ is the time of running stance; *W* is the total work on a joint during the running stance; *P*_*j*_ is the instantaneous power of a joint. The joint moment, power, and work were all divided by body weight for normalization.

#### Partial Least Squares Regression Method

Partial least squares regression (PLSR) is a regression mathematical modeling approach applied to multiple independent variables to various dependent variables (responses Y) and multiple independent variables (predictors X) to a single dependent variable (Wu et al., 2014). Partial least squares regression (PLSR) was used to compare four predictors, including initial joint angle and joint motion of the knee and ankle, and six responses, including positive and negative joint work, total joint work of the knee, and the ankle. The predictive variables included initial ankle joint angle (X1), initial knee joint angle (X2), range motion of the ankle (X3), and range motion of the knee (X4). The response variables included ankle positive work (Y1), ankle negative work (Y2), total work of the ankle (Y3), knee positive work (Y4), knee negative work (Y5) and total work of the knee (Y6).

Data standardization processing was carried out on the original data matrix X, Y to facilitate the use of formulas in subsequent operations and to express the corresponding data while reducing the error. The corresponding matrix was obtained after processing. Considering a (N×K) matrix of the mean-centered input space (X) and a (N×J) matrix of the mean-centered output space (Y), where K is the number of independent variables (factors) per observation (4 joint angles), J is the number of dependent variables per observation (6 joint work), and N is the number of the observations (50 training samples from the total of runners in this study) and subscript L is the number of components. **P** matrix and **Q** matrix are the so-called loading matrices, **E** and **F** are the residual matrices. T and U are the projection matrices. PLSR method decomposes the **X** and **Y** matrices into a bilinear structural model, consisting of a linear combination of the score and the loading matrix.

$$X_{\mathrm{NK}}=T_{\mathrm{NL}}\cdot P_{\mathrm{KL}}^{T}+E_{\mathrm{NK}}$$

$$Y_{\mathrm{NJ}}=U_{\mathrm{NL}}\cdot Q_{\mathrm{JL}}^{T}+F_{\mathrm{NJ}}$$

Step 1 The original data matrix X, Y will be normalized to facilitate the expression of the corresponding data by the formula in the subsequent operation while reducing the error between data volumes, and the corresponding matrix will be obtained after processing. The **E**_**NK**_ is the corresponding matrix for **X**_**NK**_ and **F**_**NJ**_ is the corresponding matrix for **Y**_**NJ**_ for repeated iterative operations(Xu et al., 2020).

Step 2 After obtaining the corresponding normalized matrix, the corresponding components need to be extracted. In this regression PLSR model, the number k principal components extracted for modeling is determined by the cross-validity test. Thus, H principal components are extracted, where Y_j_ is the *j*th dependent variable. The squared sum of the prediction error is shown in the following equation, where p is the total number of reaction factors:

$$\mathrm{PRESS}\left(H\right)\sum_{i=k}^{p}\mathrm{PRES}S_{j}\left(H\right)$$

The squared sum of errors of the dependent variable set Y is:

$$\mathrm{SS}\left(H\right)\sum_{j=k}^{p}SS_{j}\left(H\right)$$

According to the principal component analysis, the corresponding components should satisfy PRESS (H) while it reaches the minimum value. Generally, PRESS (H) is larger than SS (H), While SS (H) is lesser than SS (H-1). Consequently, the smaller PRESS(H)/SS(H-1) is the better. The limit value is commonly set as 0.05 (29,32).

$$Q_{H}^{2}=1-\mathrm{PRESS}\left(H\right)/\mathrm{SS}\left(H-1\right)\;=1-0.952=0.0975$$

For this reason, the model meets the accuracy requirement when the cross validity *Q* ^*2*^_*H*_ < 0.0975, while the extraction of components is stopped.

This PLSR algorithm model (Version: R2019a, The MathWorks, Natick, MA, United States) uses 80% of the data set sample size as the training set, and 20% sample size as the test set. Firstly, the training set is cross-checked by leave-one-out cross-validation analysis. Second, after cross-checking the model’s training set, the new data set was used to verify the model. The average X_ave_, maximum X_max_, minimum X_min_, the difference between the maximum and minimum X_dif_ (X_max_-X_min_) of each predictive variable is shown in Table 1. The incremental perturbation action of a predictor variable was taken to X_min_ - 10%X_dif_, X_min_, X_min_ + 10%X_dif_, X_min_ + 20%X_dif_, X_min_ + 30%X_dif_, X_min_ + 40%X_dif_, X_min_ + 50%X_dif_, X_min_ + 60%X_dif_, X_min_ + 70%X_dif_, X_min_ + 80%X_dif_, X_min_ + 90%X_dif_, X_max_, X_max_ + 100%X_dif_ in Table 2.

**TABLE 1:** The average value (X_ave_), maximum value (X_max_), minimum value (X_min_) and the difference between the maximum and minimum values (X_dif_) of predictive variables X.

X	X1 (ankle IC)	X2 (knee IC)	X3 (ROM ankle)	X4 (ROM knee)
X_ave_	8.21	19.22	43.82	27.01
X_max_	16.58	29.85	80.41	37.51
X_min_	1.97	8.44	22.53	18.34
X_dif_	14.61	21.41	57.88	19.17

**TABLE 2:** The predictors of each predictive variable.

X	X1 (ankle IC)	X2 (knee IC)	X3 (ROM ankle)	X4 (ROM knee)
X_min_-10%X_dif_	1.78	7.60	20.27	16.51
X_min_	1.97	8.44	22.53	18.34
X_min_+10%X_dif_	3.44	10.58	28.31	20.26
X_min_+20%X_dif_	4.90	12.73	34.10	22.18
X_min_+30%X_dif_	6.36	14.87	39.89	24.09
X_min_+40%X_dif_	7.82	17.01	45.68	26.01
X_min_+50%X_dif_	9.28	19.15	51.47	27.93
X_min_+60%X_dif_	10.74	21.29	57.25	29.84
X_min_+70%X_dif_	12.20	23.43	63.04	31.76
X_min_+80%X_dif_	13.66	25.57	68.83	33.68
X_min_+90%X_dif_	15.12	27.71	74.62	35.59
X_max_	16.58	8.44	80.41	37.51
X_max_+110%X_dif_	33.16	16.89	160.81	75.02

Note: X: predictor variables, X1: initial ankle angle, X2: initial knee angle, X3: range motion of ankle angle, X4: range motion of knee angle.

Note: X: ^∗^significant difference between pre-fatigue running and post-fatigue (p ≤ 0. 05).

### Statistical Analysis

Statistical analyses were performed to determine significant differences in ankle and knee joint work, joint angle, joint moment, and joint power during the stance phase. Shapiro–Wilk’s tests were performed for normal distribution. We used the paired *t*-test in SPSS 23.0 (SPSS Inc., Chicago, IL) to assess data differences for kinematic and kinetic parameters. The significance alpha level was set to 0.05. One-dimensional, one-way repeated measures Statistical Parametric Mapping (SPM) (a = 0.05) was used to assess differences in joint angle, joint moment, and joint power throughout the running stance.

## Result

**FIGURE 1:**
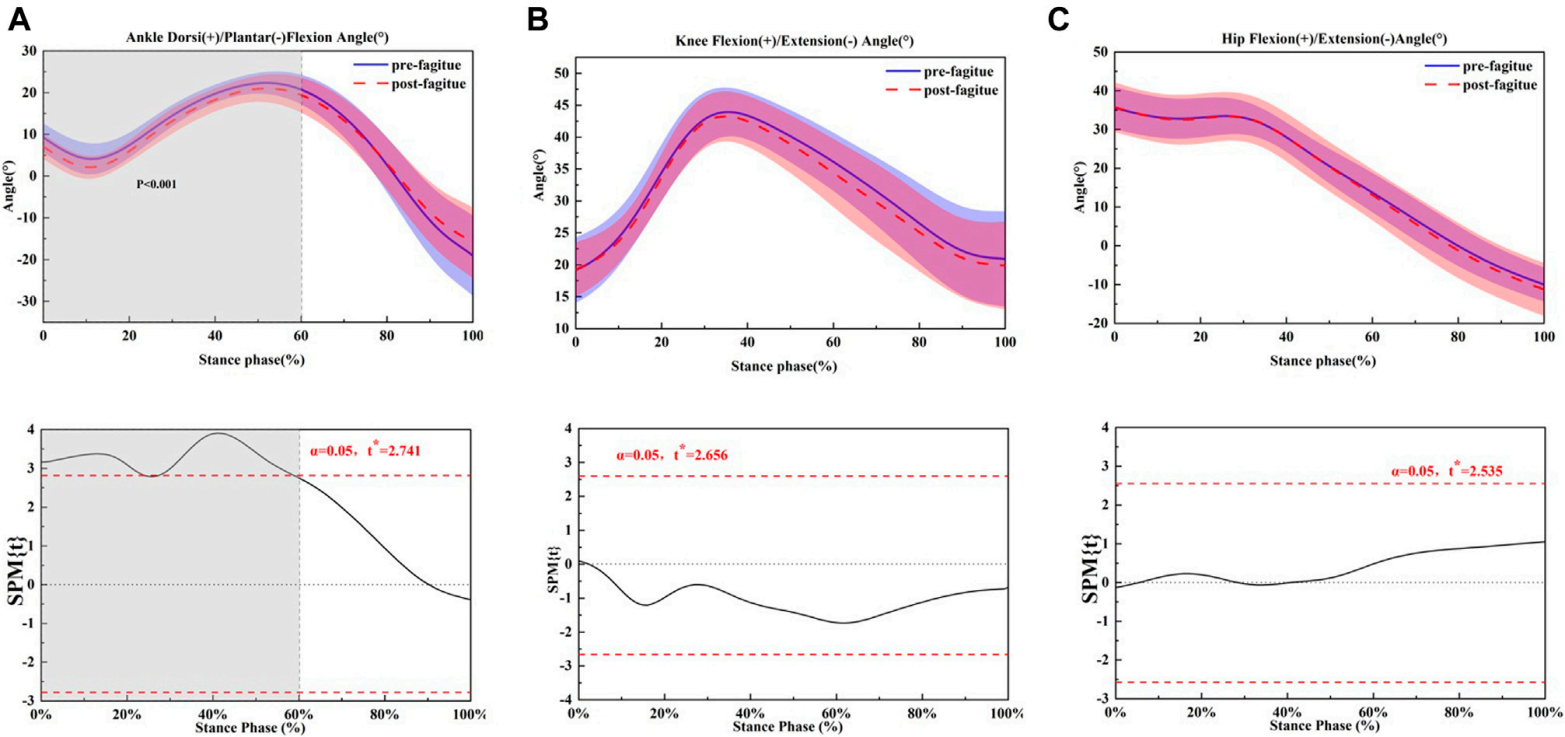
Comparing the mean values of ankle, knee and hip joint angle from all participants between fatigue conditions (pre-fatigue; post-fatigue). *p ≤ 0.05.

**FIGURE 2:**
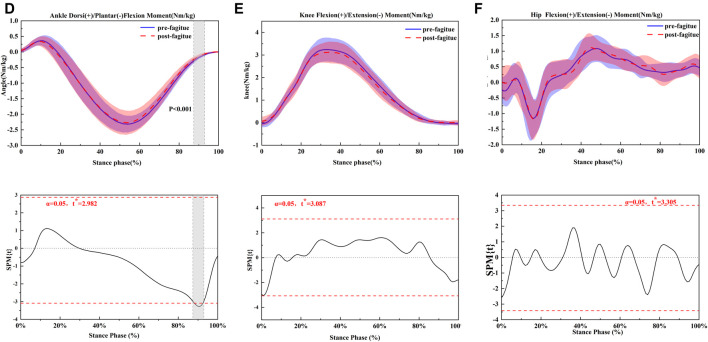
Comparing the mean values of ankle, knee and hip joint moment from all participants between fatigue conditions (pre-fatigue; post-fatigue). *p ≤ 0.05.

**FIGURE 3:**
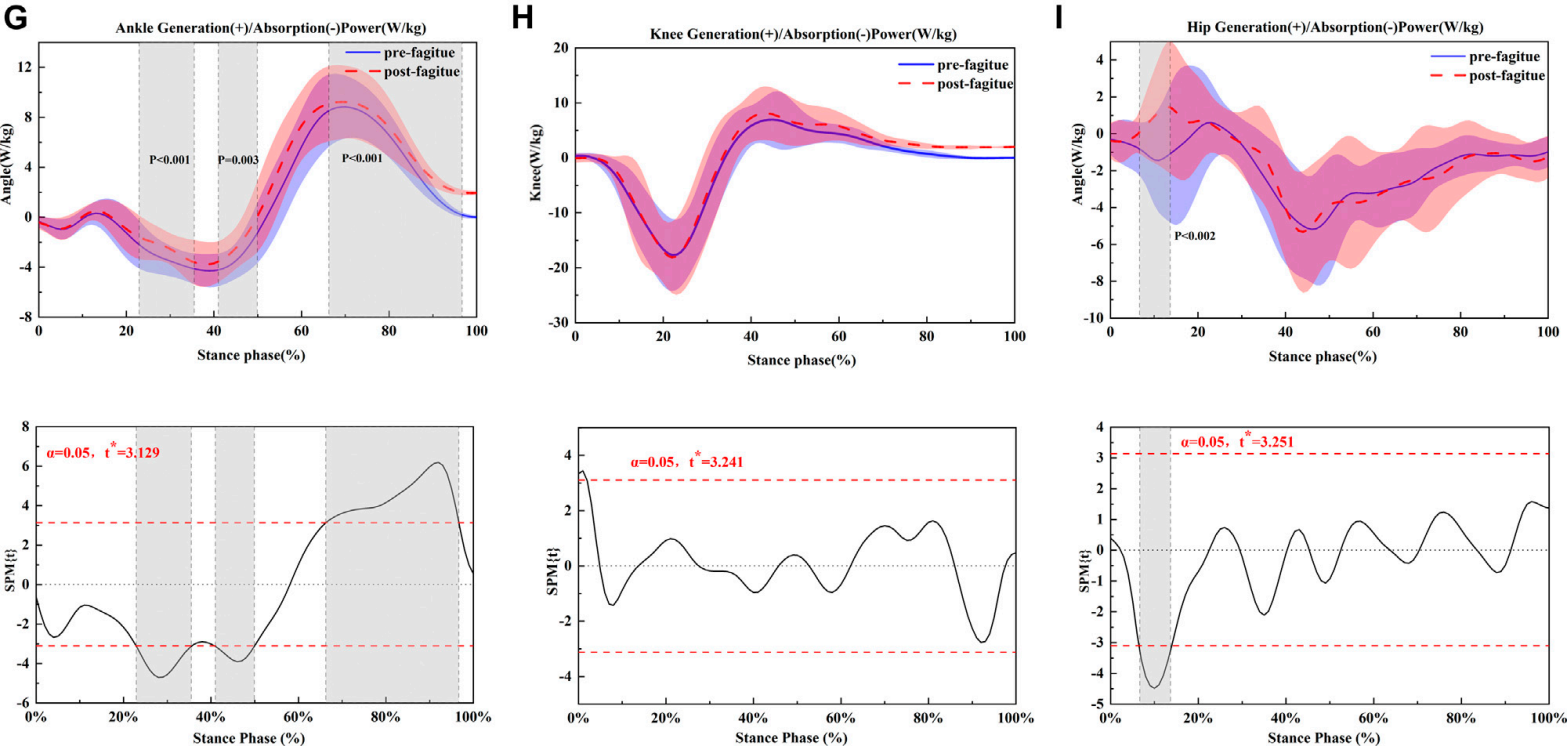
Comparing the mean values of ankle, knee and hip joint power from all participants between fatigue conditions (pre-fatigue; post-fatigue). *p ≤ 0.05.

The joint kinematics and kinetics of ankle, knee, and hip of pre-post fatigue running are shown in Figures 1–3 and Table 3; Table 4; Table 5, respectively.

**TABLE 3:** Lower extremity joint kinematics pre-fatigue running and post-prolonged fatigue running (x ± SD).

Variables	Pre-fatigue	Post-fatigue	*p*-value
Ankle IC (◦)	9.23 ± 3.44	7.19 ± 3.00	0.002*
Max dorsiflexion angle (◦)	22. 20.66 ± 3.31	20.66 ± 3.31	0.001*
Max plantarflexion angle (◦)	−19.16 ± 9.43	−18.52 ± 8.24	0.513
Range of ankle joint motion (◦)	41.74 ± 8.28	39.18 ± 13.00	0.023*
Knee IC (◦)	19.17 ± 5.12	19.27 ± 4.24	0.902
Max flexion angle (◦)	44.13 ± 3.87	43.54 ± 3.83	0.271
Range of knee joint motion (◦)	27.23 ± 4.28	26.78 ± 4.29	0.511
Max hip flexion angle (◦)	35.87 ± 5.24	36.15 ± 6.47	0.693
Max hip extension angle (°)	−9.98 ± 4.39	−11.26 ± 6.86	0.026*
Range of hip joint motion (◦)	45.86 ± 4.03	47.41 ± 4.49	0.001*

Note: ^**∗**^significant difference between pre-fatigue running and post-fatigue running (*p* ≤ 0. 05).

IC: initial contact angle.

**TABLE 4:** Lower extremity joint moment, power pre-fatigue running and post-prolonged fatigue running (x ± SD).

Variables	Pre-fatigue	Post-fatigue	*p*-value
Max ankle PF moment (Nm/kg)	-2.34 ± 0.07	−2.30 ± 0.39	0.337
Max ankle DF moment (Nm/kg)	0.42 ± 0.12	0.40 ± 0.14	0.385
Max knee flexion moment (Nm/kg)	3.34 ± 0.51	3.24 ± 0.41	0.093
Max hip flexion moment (Nm/kg)	1.37 ± 0.44	1.38 ± 0.36	0.885
Max hip extension moment (Nm/kg)	−1.58 ± 0.56	−1.57 ± 0.42	0.982
Max ankle positive power (Watt/kg)	9.42 ± 2.44	8.74 ± 2.89	0.034*
Max ankle negative power (Watt/kg)	−5.27 ± 1.13	−5.43 ± 1.71	0.535
Max knee positive power (Watt/kg)	9.48 ± 3.97	9.91 ± 4.53	0.455
Max knee negative power (Watt/kg)	−21.45 ± 6.02	−23.65 ± 6.43	0.050*
Max hip positive power (Watt/kg)	2.63 ± 2.24	3.61 ± 2.74	**0.045**
Max hip negative power (Watt/kg)	−7.80 ± 2.73	−7.43 ± 3.90	0.521

Note: ^**∗**^significant difference between pre-fatigue running and post-fatigue running (*p* ≤ 0. 05).

**TABLE 5:** Lower extremity joint work pre-fatigue running and post-prolonged fatigue running (x ± SD).

Variables	Pre-fatigue	Post-fatigue	*p*-value
Ankle positive work (J/kg)	0.63 ± 0.17	0.53 ± 0.17	0.044*
Ankle negative work (J/kg)	−0.32 ± 0.08	−0.33 ± 0.08	0.733
Ankle total work (J/kg)	0.31 ± 0.21	0.25 ± 0.16	0.046*
Knee positive work (J/kg)	0.60 ± 0.16	0.55 ± 0.16	0.273
Knee negative work (J/kg)	−0.78 ± 0.21	−0.81 ± 0.21	0.223
Knee total work (J/kg)	0.28 ± 0.17	0.26 ± 0.18	0.543
Hip positive work (J/kg)	0.06 ± 0.05	0.09 ± 0.06	0.050*
Hip negative wok (J/kg)	−0.57 ± 0.18	−0.55 ± 0.28	0.590
Hip total work (J/kg)	−0.51 ± 0.20	−0.46 ± 0.28	0.203

### Kinematic Variables

Ankle dorsiflexion angle at initial contact significantly decreased when the pre-fatigue condition and post-prolonged running condition were compared (*p* = 0.002) (Figure 1A). The maximum dorsiflexion angle was also significantly greater than post-prolonged running in the sagittal ankle plane. Due to the dorsiflexion angle decrease, the ROM of the ankle was significantly larger after prolonged running (*p* = 0.001). There were no differences present in the knee flexion angle and in ROM regarding the sagittal plane. At the hip fatigue, prolonged running had a more significant effect on the max hip extension, and the max hip extension angle was decreased when all participants after prolonged running (*p* = 0.026) (Figure 1C), but hip flexions observed no change. The range of motion of the hip was increased during the post-prolonged running (*p* = 0.001).

### Peak Torque and Power

Moderate reductions in peak positive ankle power (*p* = 0.034) (Figure 2D) were observed following the prolonged running fatigue protocol in Table 4. During running, a significantly higher knee negative power (*p* = 0.05) (Figure 2E) was found after the prolonged running fatigue protocol in Table 4. For the hip, the positive power (*p* = 0.045) (Figure 2F) was significantly increased in the fatigue and prolonged running condition, respectively (Table 4). All other peak joint moments and peak positive and negative joint powers remained unchanged following the prolonged running fatigue protocol.

### Joint Work

Relative positive ankle work was significantly increased after prolonged running (*p* = 0.044). A moderate reduction in the absolute total of ankle work was observed after prolonged running (*p* = 0.046). Relative negative knee work and knee positive work were moderately unchanged following the prolonged running fatigue protocol (Table 4). In addition, the hip positive work was significantly greater when participants after prolonged running (*p* = 0.050).

### PLSR Model

**FIGURE 4:**
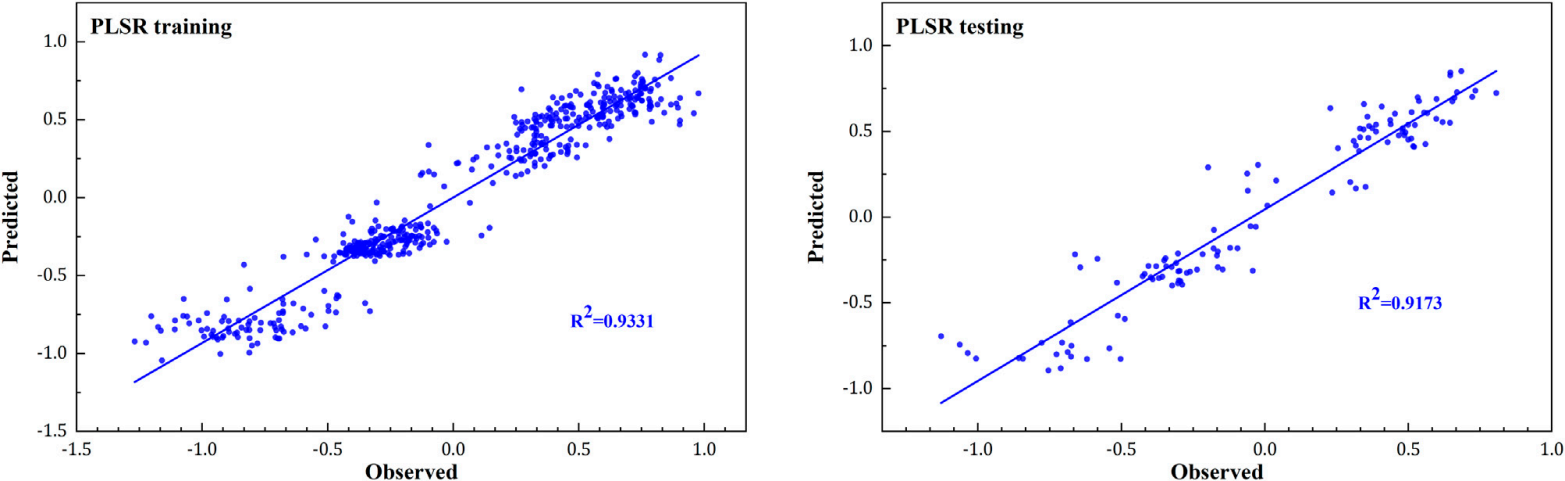
Training (left) and testing (right) accuracy of special skills assessment results of observed and predicted from the PLSR model in the female runners.

**FIGURE 5:**
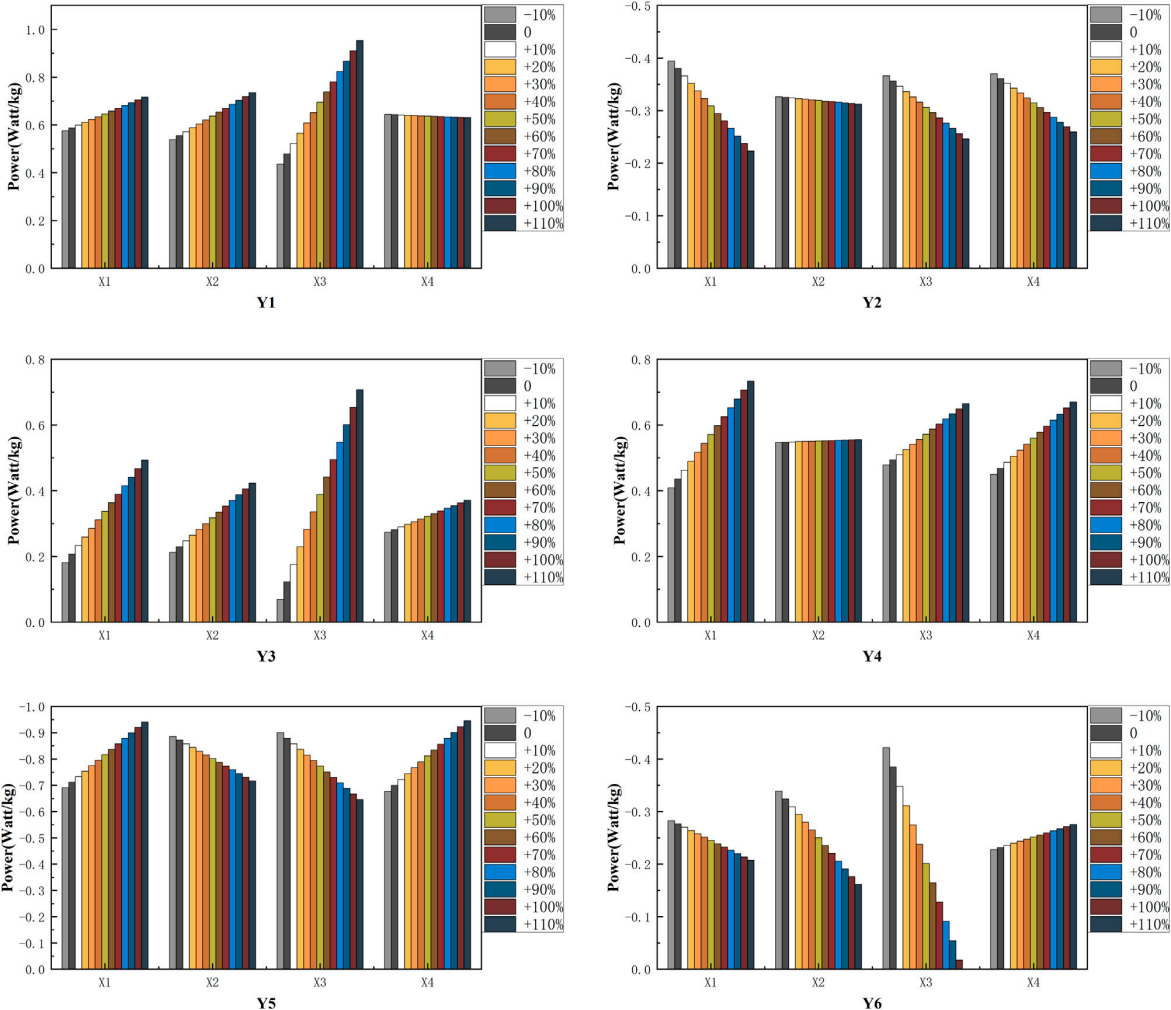
The predicted results of the response variables base on the PLSR model. Ankle positive work (Y1), ankle negative work (Y2), total work of the ankle (Y3), knee positive work (Y4), knee negative work (Y5) and total work of the knee (Y6). Initial ankle joint angle (X1), initial knee joint angle (X2), range motion of the ankle (X3), and range motion of the knee (X4).

PLSR models for female amateur runners (Figure 4) were trained separately for ankle positive work (Y1), ankle negative work (Y2), total work of the ankle (Y3), knee positive work (Y4), knee negative work (Y5) and total work of the knee (Y6). A “leave-one-out” analysis showed a response variable prediction accuracy of 93.31% for the training set and 91.73% for the test set. The results of the sensitivity analysis of the PLSR model based on the independent variable set disturbance factor are shown in Figure 5.

Under the control of other independent variable sets, we can find 1) with increased initial ankle angle, under the same conditions, the joint work for runners after fatigue running, Y2 and Y6 were reduced, and the joint work for runners after fatigue running Y1, Y3, Y4 and Y5 were increased. 2) with increased initial knee angle, under the same conditions, the joint work for runners after fatigue running Y5 and Y6 were reduced, and the joint work for runners after fatigue running Y1, Y3 were increased. 3) with the increased motion of the ankle, under the same conditions, the joint work for runners after fatigue running Y2, Y5, and Y6 were reduced, and the joint work for runners after fatigue running Y1, Y3 and Y4 were increased. 4) with the increased motion of the knee, under the same conditions, the joint work for runners after fatigue running Y2 was reduced, and the joint work for runners after fatigue running Y3, Y4, Y5 and Y6 were increased.

## Discussion

This study aimed to describe and examine the kinematic and joint mechanical parameters of female recreational runners who sustained prolonged running until reaching the point of fatigue. This study’s primary findings are the following: decreased ankle initial contact angle and range of motion of the ankle was found following fatigue running, which agrees with our hypothesis. At the hip joint, the extension angle was significantly decreased, but the motion of the hip increased in the fatigue prolonged running condition. Furthermore, skeletal joint work was significantly reduced regarding the kinetics, including positive ankle work and total ankle work. However, fatigue running resulted in increased hip positive power and hip positive work. When comparing the knee parameters, negative knee power was higher following prolonged running compared to the initial status. However, there was no significant change in the joint angle, positive power, and joint work of knee parameters in this study.

In the present study, significant fatigue effects on lower limb kinematics and kinetics were found following prolonged running as highlighted in the lower limb kinematics: pre-running and post-fatigue running presented different joint angles, joint power, and joint work change trends. A recent study has compared joint work following prolonged running and found that joint work decreased in the distal lower limb joints (Sanno et al., 2018). After prolonged running, the ankle plantar flexor muscle activity was significantly reduced, which led to joint work redistribution in the ankle joint. Our results are consistent with this finding. With the distal ankle work decreased proximally, hip power significantly increased in the fatigue running condition. Several factors were identified as the underlying mechanisms that caused joint work redistribution following fatigue-induced prolonged running. First, the initial contact angle, max dorsiflexion angle, and range of ankle motion during the stance were significantly decreased in the prolonged fatigue condition compared with the pre-fatigue condition. The decrease in ankle dorsiflexion angle will reduce the moment arm of the ankle joint. A combination of smaller dorsiflexion initial contact and a smaller range of motion of the ankle resulted in smaller positive power, and therefore more minor positive joint work and total energy dissipation at the joint. Moreover, the running distance, sex, and the runner’s ability level might affect joint work redistribution following prolonged fatigue running. During the stance of running, the energy distribution of the lower limbs follows the conservation of energy. After a fatigue prolonged running, a decrease in the work done by the distal ankle joint results in an increase in the work and power done by the hip joint. In this study finding, the hip ROM was significantly increased when runners in post-fatigue prolonged running. The study by Winter et al. is that the ROM of the hip joint increases significantly after fatigue, which also supports this result (Winter et al., 2016).

However, there was no change in knee joint work moment and angle in our study. After fatigue prolonged running, the knee flexion with no changes might be due to fatigue running not being associated with knee flexion. Accordingly, the knee was a vital joint during the running stance, absorbing shock and dissipating the ground reaction force (Zhang et al., 2000). In the current study, it has been found that knee negative power was increased in the fatigue prolonged running condition. It has also been demonstrated that there were no changes of the knee and hip joint positive work following a fatiguing treadmill running for the well-trained runners (Melaro et al., 2021).

In addition, it has been noted that following prolonged running, the muscle of the foot also undergoes modification (Christina et al., 2001), which inverts or dorsiflexes following fatigue. It has been found that prolonged fatigue running might lead to dorsiflexor fatigue, increasing the lower extremity attenuation capability to heel impacts (Duquette and Andrews, 2010). Therefore, if the dorsiflexors are fatigued, the initial dorsiflexion contact angle and max dorsiflexion angle are smaller in the fatigue condition, which was consistent with our results. The decreased positive joint work and total joint work of the ankle may result from muscular fatigue. However, we did not collect any muscle change data prior to and after the prolonged running fatigue protocol. In the future, researchers should consider muscle fatigue and investigate the relationship between joint work redistribution.

It should be noted that while the PLSR method has been used to predict plantar pressure (Mei et al., 2019b), foot posture, joint kinematics, joint moments, and joint contact forces in gait analysis (Mei et al., 2019a), this is the first study which applies PLSR models to correlate initial angles with prolonged running fatigue joint work in amateur female runners.

Our model observed that the ankle angle at IC decreased in the fatigue prolonged running, and the joint work showed a high linear correlation, which is consistent with the previous study. Furthermore, with the dorsiflexion decreased, the ankle work and total ankle work were also smaller than the pre-fatigue condition (Sanno et al., 2018; Melaro et al., 2021). Moreover, after prolonged fatigue running, knee flexion may increase more than pre-running. The increased knee flexion angle will decrease the arm of the proximal joint, and the knee joint work will also be smaller than pre-running. This model can be used to analyze the angle changes after fatigued running, which can predict the redistribution and alteration of the work of the ankle and knee joint. Using this prediction model, it is possible to understand the change of work in lower limb joints following prolonged running.

There are also some limitations to our study. First, all subjects were amateur female runners, therefore we need to consider different levels of runners to compare the joint work before and after prolonged running in the future. A considerable variance existed in knee outcome measures. Additional measurement trials may help to overcome this problem. In the future, more attention needs to be paid to muscle fatigue and running economy tests. We used a subjective measure (RPE) and heart rate to rate physical fatigue following treadmill running. While these measures are accurate and valid, we cannot be 100% certain that all participants were totally exhausted at the end of the running fatigue protocol.

## Conclusion

This study shows an investigation of the changes in joint mechanics, joint kinematics, joint moments, and joint power in the lower extremity following a fatiguing treadmill run in 50 female recreational runners. A relationship between knee and ankle initial angle and joint work was developed. It was found that moderate reductions in absolute positive ankle power, total ankle energy dissipation, dorsiflexion at initial contact, max dorsiflexion angle, and range of motion of the joint ankle were observed after fatigue following prolonged running. Knee joint mechanics, joint angle, and joint power were unchanged following prolonged running. However, with the decreased ankle joint work, negative knee power, increased hip positive work, and hip positive power were increased during initial foot contact following running due to fatigue. These results suggest no proximal shift in knee joint mechanics in female recreational runners following a prolonged run. The joint work redistribution was associated with running fatigue changes. To improve running performance, long-distance runners should include ankle muscle strength training to avoid running-related injuries.

## Data Availability

The datasets generated for this study are available on request to the corresponding author.
